# Grand Challenges in Neuroinfectious Diseases

**DOI:** 10.3389/fneur.2017.00480

**Published:** 2017-09-14

**Authors:** Avindra Nath

**Affiliations:** ^1^Section of Neuroinfectious Diseases, National Institutes of Neurological Disorders and Stroke, National Institutes of Health, Bethesda, MD, United States

**Keywords:** neuroinfectious diseases, specialty grand challenge, emerging infections, lack of clinical trials, antiviral drugs, microbiome, endogenous, neuroimmune disorders

When Neurology evolved as a separate sub-specialty of Medicine, it was in large measure due to infections that affected the nervous system. This included many of the childhood infections—such as measles, mumps, rubella, tetanus, polio, rabies—and bacterial meningitis, brain abscesses, and syphilis in adults. Over the years, with major advancements in development of vaccines, antibiotics, and changes in hygiene practices, many of these infections have been well controlled, and the pattern of infections seems to be constantly evolving. However, the field of neuroinfectious diseases continues to face many major challenges. Below I highlight some of the critical areas for which major developments are needed and urge clinicians and researchers to help fill these gaps.

## Emerging Infections

Unlike other subspecialties in Neurology, in the field of neuroinfectious diseases, new clinical syndromes and disorders are emerging on a regular basis. They may occur as outbreaks in small localized regions or spread rapidly over large geographical areas. This poses multiple challenges for the health-care system, the practicing physicians, and the researchers. They may also have far reaching socioeconomic consequences. Recent examples include Zika virus, Chikungunya, West Nile, acute flaccid paralysis with enterovirus infection and Ebola virus infection. Nearly all recent epidemics have severe neurological manifestations and have spread rapidly across continents. Although these infections get much media attention in the beginning, the neurological devastation of the affected populations remains long after the cameras and light have been dimmed in these regions. This is when developing an understanding of the pathophysiology of the disease, its neurological manifestations, and developing modes of management and prevention of secondary complications is much needed. It is hard to predict what the next epidemic would be. However, neurologists should keep a close eye on some of the tick-borne illnesses such as Powassan encephalitis, Jamestown Cannon virus, and some of the equine encephalitides, which seem to be on the rise (1, 2). Neurological complications of measles and mumps are reemerging in populations that cannot be vaccinated because of being in war-torn regions or in populations that choose not to get vaccinated (3).

## Lack of Clinical Trials

Due to the acute nature of the infections, delay in establishing an etiological diagnosis and unpredictability of where these cases might emerge, clinical trials with CNS infections are far and few between. Guidelines for management are often based on experience with case series and influenced by the opinion of experts in the field. Establishing consensus statements for diagnosis and management of these syndromes is critically important. Where differences in opinion or controversies exist, one must present both sides and highlight the lack of knowledge that leads to such diverse opinions. This will likely spur interest in conducting studies to address these issues.

## Lack of Antiviral Drugs

Except for herpes viruses and HIV, there are no antiviral drugs available for the wide variety of pathogens that can cause CNS infections. Animal models, *in vitro* drug discovery programs, and human studies are necessary to make an impact on these infections. In some cases, the antimicrobial agents alone might not be sufficient, and the host response to the infection might be damaging to the CNS. Hence, studies are needed to characterize these responses and to manage them appropriately in experimental models and clinical studies. Since many of the infections occur in small outbreaks and sporadically, major drug companies have not taken on the challenge for developing such antiviral compounds. Hence, broad spectrum antiviral is necessary that may be effective against a whole class of viral pathogens (4).

## Undiagnosed Infections

Nearly 20–30% of patients with suspected CNS infections in any tertiary care facility never get an etiological diagnosis (5). The reasons for which remain unclear. Timely collection and proper handling of samples may in part be the issue. Lack of easy access to broad panels or deep sequencing to look for known and rare microbial pathogens and lack of expertise to study these infections may be to blame. Such techniques are available in research laboratories, but once new organisms have been found or techniques optimized for detection of known pathogens, it could be adapted for general use. A challenge in translating discoveries made in research labs to the clinic is often the lack or expertise and the laborious task of validation of the biomarker to where is can produce reliable and consistent results. This may require collaborations with diagnostic laboratories and input from the Federal Drug Administration in the United States and similar regulatory agencies elsewhere.

## Microbiome and Endogenous Viruses

Much attention in recent years has been given to the microbiome in the gut and other mucosal surfaces. The composition of the microbiome varies in different disease states, and it has been suggested that it may drive the pathophysiology of several neuroinflammatory diseases and influence the innate immune responses in neurodegenerative diseases (6). It is still unclear if there are specific microorganisms that can drive the response or if the delicate balance of microorganisms is the critical feature. Based on these findings, intervention studies are being conducted in patients with multiple sclerosis and autism to see if manipulation of the microbiome can influence the disease course (7).

While the role of protein aggregates such as prions is well established in the transmission of Creutzfeldt–Jakob disease and its variants, recent studies have been investigating a similar role for protein aggregates in the transmission of diseases such as Alzheimer’s disease, Parkinson’s disease, and multiple system atrophy (8). Interesting developments in manipulating these protein aggregates are expected in the near future. It is also becoming apparent that the human chromosome encodes for several retroviral sequences that were acquired through the process of evolution. Many of these sequences are defective or silenced by epigenetic changes and hence have been largely ignored by researchers. Recent studies in patients with multiple sclerosis, schizophrenia, and amyotrophic lateral sclerosis have shown activation of these viral elements, which may play a role in the pathophysiology of these diseases (9, 10). Besides retroviruses, in nearly 1% of the population, the human chromosome has an integrated copy of human herpes virus 6 (11). The role of this integrated virus in disease states is not clear.

## Role of Infections in Neuroimmune Disorders

It has been shown that viral infections can trigger immune disorders. For example, it is well established that acute disseminated encephalomyelitis, transverse myelitis, and Gillian–Barré syndrome are most often triggered by a viral infection. The underlying mechanisms are more complex. Molecular mimicry between the viral pathogen and host antigens is often considered to be an explanation for these manifestations. However, the precise antigens are not well defined, and ability to reproduce these manifestations in animal models has been difficult. Recent studies show that herpes simplex virus type-1 encephalitis can trigger an autoimmune encephalitis due to NMDA receptor antibodies (12). These phenomena are not restricted to viral infections. It is well known that streptococcal infections can trigger an autoimmune phenomenon affecting the basal ganglia and is termed Sydenham’s chorea (13). Recently, a parasitic infection, *Onchocerca volvulus*, has been shown to trigger an epileptic disorder called Nodding syndrome in endemic regions (14). It this likely that there are many other yet to be discovered autoimmune disorders that are triggered by a wide variety of pathogens. In these patients, controlling the underlying infection alone may not be sufficient, but modulation of the immune response may also be necessary. Clinical trials are needed to guide the management of such patients.

In summary, infections of the nervous system have caused devastating human diseases affecting large populations but pose some of the most unique challenges in medicine. Current measures for diagnosis and treatment are suboptimal, prevention strategies are poor and major investment of human capital and resources are necessary if we have any hope of conquering these illnesses.

## Author Contributions

The author confirms being the sole contributor of this work and approved it for publication.

## Conflict of Interest Statement

The author declares that the research was conducted in the absence of any commercial or financial relationships that could be construed as a potential conflict of interest.
